# Drivers of Seasonal Change of Avian Communities in Urban Parks and Cemeteries of Latin America

**DOI:** 10.3390/ani14243564

**Published:** 2024-12-10

**Authors:** Lucas M. Leveau, Lucia Bocelli, Sergio Gabriel Quesada-Acuña, César González-Lagos, Pablo Gutierrez Tapia, Gabriela Franzoi Dri, Carlos A. Delgado-V, Alvaro Garitano-Zavala, Jackeline Campos, Yanina Benedetti, Rubén Ortega-Álvarez, Anotnio Isain Contreras-Rodríguez, Daniela Souza López, Carla Suertegaray Fontana, Thaiane Weinert da Silva, Sarah S. Zalewski Vargas, Maria C. B. Toledo, Juan Andres Sarquis, Alejandro Giraudo, Ada Lilian Echevarria, María Elisa Fanjul, María Valeria Martínez, Josefina Haedo, Luis Gonzalo Cano Sanz, Yuri A. Peña Dominguez, Viviana Fernandez-Maldonado, Veronica Marinero, Vinícius Abilhoa, Rafael Amorin, Juan Fernando Escobar-Ibáñez, María Dolores Juri, Sergio R. Camín, Luis Marone, Augusto João Piratelli, Alexandre G. Franchin, Larissa Crispim, Federico Morelli

**Affiliations:** 1 Departamento de Ecología, Genética y Evolución, Facultad de Ciencias Exactas y Naturales, Universidad de Buenos Aires—IEGEBA (CONICET—UBA), Ciudad Universitaria, Pab 2, Piso 4, Buenos Aires 1426, Argentina; 2 Laboratorio de Ecología Urbana, Vicerrectoría de Investigación, Universidad Estatal a Distancia, San José 2050, Sabanilla, Costa Rica; 3 Departamento de Ciencias, Facultad de Artes Liberales, Universidad Adolfo Ibáñez, Santiago 8320000, Chile; cesar.glagos@gmail.com; 4 Center of Applied Ecology and Sustainability (CAPES), Santiago 7820244, Chile; 5 Geobiota Consultores, Avenida Andrés Bello 2325, Piso 12, Santiago 7500000, Chile; 6 Department of Wildlife, Fisheries, and Conservation Biology, University of Maine, 5755 Nutting Hall, Room 244, Orono, ME 04469-5755, USA; 7 Programa de Ecología, Facultad de Ciencias y Biotecnología, Universidad CES, Calle 10A 22-04, Medellín 050021, Colombia; 8 Instituto de Ecología, Facultad de Ciencias Puras y Naturales, Universidad Mayor de San Andrés, La Paz 8635, Bolivia; agaritanozavala@umsa.bo; 9 Independent Researcher, Av. Sánchez Lima 900, Torre Altavista 14F, La Paz, Bolivia; 10 Faculty of Environmental Sciences, Community Ecology & Conservation, Czech University of Life Sciences Prague, Kamýcká 129, CZ-165 00 Prague, Czech Republic; ybenedetti73@gmail.com (Y.B.); fmorellius@gmail.com (F.M.); 11 Investigadoras e Investigadores por México del CONACYT, Dirección Regional Occidente, Comala 28454, Mexico; rubenortega.al@gmail.com; 12 Centro de Cultura Ambiental, Acuexcomatl, SEDEMA, Ciudad de México 16610, Mexico; 13 North American Birds Conservation Initiative, CONABIO, Liga Periférico-Insurgentes Sur No. 4903, Parques del Pedregal, Ciudad de México 14010, Mexico; 14 Center of Ecology, Universidade Federal do Rio Grande do Sul, Porto Alegre 91540-000, RS, Brazil; xolmis@gmail.com; 15 Museu de Ciências e Tecnologia, Programa de Pós-Graduação em Ecologia e Evolução da Biodiversidade, Pontifícia Universidade Católica do Rio Grande do Sul, Av. Ipiranga 6681, Prédio 40 Sala 110 B, Porto Alegre 90619-900, RS, Brazil; thaianews@gmail.com; 16 Laboratório de Ornitologia, Museu de Ciências e Tecnologia, Pontifícia Universidade Católica do Rio Grande do Sul, Porto Alegre 90619-900, RS, Brazil; sarah_szv@hotmail.com; 17 Laboratório de Ecologia, Instituto Básico de Biociências, Universidade de Taubaté Curso de Pós-Graduação em Ciências Ambientais, Taubaté 12020040, SP, Brazil; mceciliabt@gmail.com; 18 Instituto Nacional de Limnología (Consejo Nacional de Investigaciones Científicas y Técnicas–Universidad Nacional del Litoral), Ciudad Universitaria, Santa Fe 3000, Argentina; juandres.sarquis@gmail.com (J.A.S.); alejandrogiraudo@hotmail.com (A.G.); 19 Instituto de Vertebrados—Zoología—Fundación Miguel Lillo, Miguel Lillo 251 San Miguel de Tucumán, Tucumán CP 4000, Argentina; adaechevarria@gmail.com (A.L.E.); mefanjul@lillo.org.ar (M.E.F.); mvmartinez@lillo.org.ar (M.V.M.); 20 Instituto de Ecología Regional (CONICET—UNT), Tucumán T4107, Argentina; johaedo@gmail.com; 21 Museo de Historia Natural de la Universidad Nacional de San Agustín de Arequipa, Arequipa 04001, Peru; lugocasan@gmail.com (L.G.C.S.); yuriadais123@gmail.com (Y.A.P.D.); 22 Centro de Investigaciones de la Geósfera y la Biósfera-CONICET, Facultad de Ciencias Exactas, Físicas y Naturales, Universidad Nacional de San Juan (UNSJ), Complejo Universitario “Islas Malvinas”, Av. Ignacio de la Roza 590 (O), Rivadavia J5402DCS, Argentina; vivifernandezm@unsj-cuim.edu.ar (V.F.-M.); veronicamarinero@gmail.com (V.M.); 23 Museu de História Natural Capão da Imbuia, PMC Rua Prof. Benedito Conceição, 407, Curitiba 82810-080, PR, Brazil; vabilhoa@uol.com.br (V.A.); amorin.rafael@hotmail.com (R.A.); 24 Doctorado en Ciencias de la Sustentabilidad, Universidad Rosario Castellanos de la Ciudad de México, Ciudad de México 07969, Mexico; juanfer.escobarib@gmail.com; 25 Gnósis—Naturaleza con Ciencia, A.C., Guadalajara 45239, Mexico; 26 IAMRA, Universidad Nacional de Chilecito, Chilecito 5360, Argentina; mdjuri@gmail.com; 27 ECODES, Grupo de Investigación en Ecología de Comunidades de Desierto, IADIZA-CONICET, Mendoza y Facultad de Ciencias Exactas y Naturales, UNCuyo, Mendoza CP 5500, Argentina; srcamin@mendoza-conicet.gob.ar (S.R.C.); lmarone@mendoza-conicet.gob.ar (L.M.); 28 Departamento de Ciências Ambientais, CCTS, Universidade Federal de São Carlos, Rodovia João Leme dos Santos, Km 110, Itinga, Sorocaba 18052-780, SP, Brazil; piratelli@ufscar.br (A.J.P.); agfranchin@hotmail.com (A.G.F.); laricrispim5@gmail.com (L.C.)

**Keywords:** biogeography, birds, climate, macroecology, Neotropical Region, urbanization

## Abstract

Urban parks and cemeteries constitute hot spots of bird diversity in urban areas. However, the seasonal dynamics of their bird communities have been scarcely explored at large scales. This study aims to analyze the drivers of urban bird assemblage seasonality in urban parks and cemeteries comparing assemblages during breeding and non-breeding seasons in the Neotropical Region. Since cemeteries have less human disturbance than urban parks, we expected differences in bird community seasonality between habitats. The seasonal change of species composition was partitioned into species turnover and nestedness. At large scales, the seasonal change of species composition was positively related to temperature seasonality and was higher in the Northern Hemisphere. At the landscape scale, the seasonal change of composition decreased in sites located in the most urbanized areas. At the local scale, sites with the highest habitat diversity and pedestrian traffic had the lowest seasonal change of composition. The species turnover was higher in the Northern Hemisphere, augmented with increasing annual temperature range, and decreased in urban parks. The species nestedness was positively related to habitat diversity. Our results showed that a multi-scale framework is essential to understand the seasonal changes of bird communities. Moreover, the two components of seasonal composition dissimilarity showed contrasting responses to environmental variables. Although the surrounding urbanization lowered the seasonal dynamics of urban green areas, cemeteries seem to conserve more seasonal changes than urban parks. Thus, urban cemeteries help to conserve the temporal dynamics of bird communities in cities.

## Simple Summary

Urban parks and cemeteries constitute hot spots of bird diversity in urban areas. However, the seasonal dynamics of their bird communities have been scarcely explored at large scales. This study aims to analyze the drivers of urban bird assemblage seasonality in urban parks and cemeteries comparing assemblages during breeding and non-breeding seasons in the Neotropical Region. At large scales, the seasonal change of species composition was positively related to temperature seasonality and was higher in the Northern Hemisphere. At the landscape scale, the seasonal change of composition decreased in sites located in the most urbanized areas. At the local scale, sites with the highest habitat diversity and pedestrian traffic had the lowest seasonal change of composition. The species turnover was higher in the Northern Hemisphere, augmented with increasing annual temperature range, and decreased in urban parks. The species loss between breeding and non-breeding seasons was negatively related to habitat diversity. Although the surrounding urbanization lowered the seasonal dynamics of urban green areas, cemeteries seem to conserve more seasonal changes than urban parks. Thus, urban cemeteries help to conserve the temporal dynamics of bird communities in cities.

## 1. Introduction

The analysis of the temporal variation of species assemblages is one of the main objectives of community ecology [1]. In recent years, several authors have highlighted the need for more research regarding the species composition change over time, which is defined as temporal beta diversity [2]. The component of temporal beta diversity has received scarce attention and, in general, authors considered mainly the interannual change of species composition [3]. However, the focus on intra-annual changes in species composition, such as seasonal changes, is extremely relevant for understanding how communities will respond to impacts produced by land use and climate change.

In the case of birds, species assemblages show annual repeatable changes in composition due to the seasonality of environmental conditions, such as duration of daytime, temperature, and precipitation [4,5,6]. The seasonal change of species composition was analyzed along latitudinal gradients, showing that resource fluctuations determine the proportion of migrant species in assemblages [7,8,9,10]. On the other hand, the seasonal change of composition may also be positively related to the annual pool of species recorded in a site [11,12].

Studies that measured assemblage seasonality generally were based on the proportion of latitudinal migrants [9,13]. However, the bird assemblages’ seasonality can also be due to fluctuations of longitudinal migrants, altitudinal migrants, or partial migrants [14,15,16]. Therefore, analyses that consider the seasonal change of the entire assemblage are necessary to gain a more realistic picture of seasonal beta diversity. Moreover, studies that evaluated the seasonal change of bird assemblages at large scales used data obtained from independent local studies, bird distributional maps, or citizen science data such as eBird [9,12,17]. These types of data preclude analyzing local-scale variables unexplored until now, such as habitat diversity, which is thought to decrease the seasonal change of bird communities [5,18,19]. Habitats with several vegetation strata may increase the amelioration of microclimatic factors [20], then buffering the seasonal change of bird resources [5].

Green areas are urban habitats dominated by vegetation, which are fundamental for biodiversity conservation within cities [21]. The role of urban parks as hot spots of bird diversity has been largely documented [21,22,23]. In addition, cemeteries constitute a type of green area within cities that also have a great potential for biodiversity conservation [24,25,26]. Several studies have found that bird species richness in cemeteries is similar to that in urban parks [27,28,29]. However, the study of bird communities in cemeteries has been performed mainly in Europe and North America [25,27,28,30], although recent studies have been published in South America [29,31]. Moreover, seasonal dynamics of bird species composition in cemeteries have been scarcely analyzed [31].

Urban areas are thought to decrease the seasonal change of bird communities due to the constant food supply for generalist resident species and the decrease in food availability for migrant species that feed on insects [17,32,33,34]. Moreover, residential areas composed of houses with wide yards and high habitat diversity are related to an annual stabilization of food resources and a decrease in the bird assemblage seasonality compared to rural areas [35]. The role of environmental buffering affecting large-scale variations of the seasonal beta-diversity in urban bird assemblages has not been evaluated yet.

**Figure 1:**
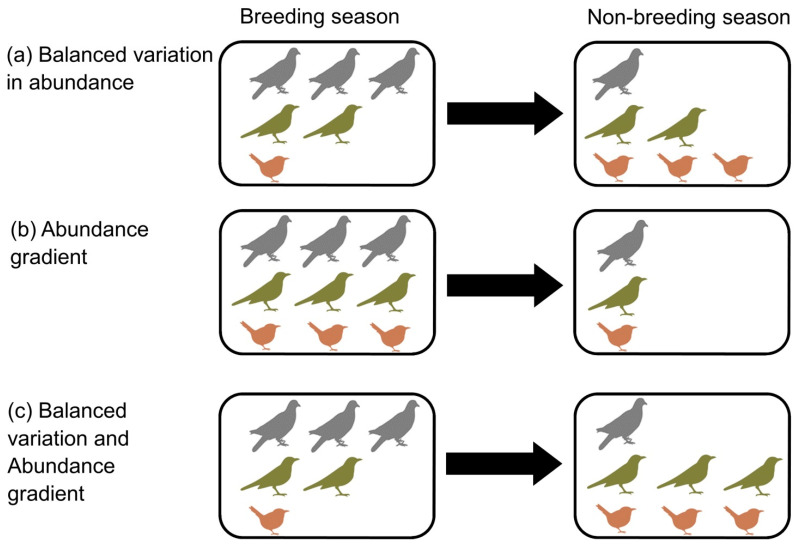
Schematic representation of (**a**) balanced variation in abundance, (**b**) abundance gradient, and (**c**) the presence of balanced variation and abundance gradient. In (**a**), some species lose individuals between seasons, whereas others gain individuals in the same proportion. In (**b**), all species lose individuals in the same proportion between breeding and non-breeding seasons. In (**c**), two species gain individuals whereas the other species lose individuals. Other hypothetical situations where species appear or disappear between seasons are also possible (see [36]).

The seasonal beta diversity can be partitioned into two components ([36], Figure 1): (1) balanced variation in abundance, which describes the turnover of some individuals of one species by the same amount of individuals of other species between seasons (Figure 1a); and (2) abundance gradients, which describes the nestedness or loss of individuals of different species between seasons (Figure 1b). Moreover, seasonal changes in bird composition may be due to the simultaneous patterns of balanced variation and abundance gradient (Figure 1c). For example, in the hypothetical case of species dissimilarity between seasons in a site is 0.50, 0.25 can be attributed to balanced variation in abundance, whereas the other 0.25 can be attributed to abundance gradients. The partitioning of the seasonal dissimilarity in turnover and nestedness components can bring more insights into the underlying processes shaping bird communities throughout the year. The role of these two components on the large-scale variation of the seasonal beta diversity of bird assemblages still remains to be studied.

In this study, we present the analyses of a large-scale coordinated survey of bird assemblages in urban parks and cemeteries of the Neotropical Region. Our study aimed to determine the influence of large-scale, landscape, and local factors on the seasonal composition dissimilarity. At a large scale, we expected that bird assemblages located in areas with the highest annual climatic seasonality and near North America would have the highest seasonal change in bird composition, given that bird assemblages of the northern hemisphere part of the Neotropical Region have a higher proportion of migratory species than the Southern American bird assemblages [37]. At the landscape scale, we expected that urban parks and cemeteries located in the most urbanized areas of the city would have the lowest seasonal bird composition change compared to those located in less urbanized areas [38,39]. On the local scale, we expected that parks and cemeteries with the highest habitat diversity would have the lowest seasonal change of composition [5]. Finally, we expected that components of beta diversity, such as nestedness and turnover, would vary differently with environmental variables.

## 2. Materials and Methods

**Figure 2:**
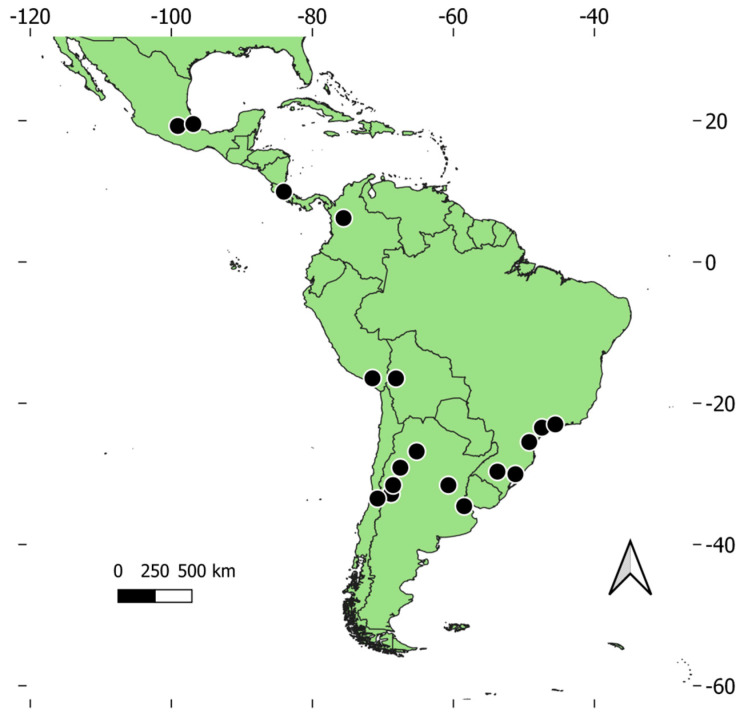
Location of study sites (black dots) in Latin America.

This study was carried out in parks and cemeteries of 18 cities in the Neotropics encompassing eight countries (Table S1, Figure 2). The absolute latitude of sites ranged between 6° (Medellín, Colombia) and 34° (Buenos Aires, Argentina), whereas altitude varied from 10 masl (Porto Alegre, Brazil) to 3625 masl (La Paz, Bolivia). In each city, cemeteries were selected and then parks with a similar location and size within the city were also chosen (see Leveau [29]). This procedure resulted in a total of 36 cemeteries and 37 parks surveyed as our sampling units (Table S1, Figure 2).

### 2.1. Bird Surveys

We surveyed birds for 10 min using point counts within a radius of 100 m, and points were at least 200 m apart [40]. The location of points was determined by a stratified design, locating points in each microhabitat (wooded areas, lawned areas, etc., [29]). Surveys were conducted during the first four hours after sunrise on weekdays only under favorable conditions (i.e., avoiding rainy and windy days) [40]. Only birds that used the sites, eating or perching, were counted, avoiding birds that flew high above. To ensure temporal replication, the same observer visited each point twice during the breeding season (spring) and twice during the non-breeding (fall) season. In the Southern Hemisphere, the breeding season corresponded to surveys during the first 2 weeks of October and the last week of November or the first week of December [29]. The non-breeding season corresponded to surveys during April, May or June. In the Northern Hemisphere, October–December corresponded to the non-breeding season, whereas April–June was the breeding season [29]. Although sites near the Equator are thought to be aseasonal, the breeding peak in sites north of the Equator (Guatemala, Costa Rica) occurs in April [41], whereas the breeding peak occurs during November [42] or between December and April [43] in areas south of the Equator (like Ecuador and Peru). Point count number per site ranged between 1 and 11. Parks and cemeteries smaller than 3 ha generally had one point, whereas the number of points increased in larger sites.

### 2.2. Predictor Variables

A total of 11 environmental variables were analyzed, including local, landscape, and large-scale characteristics (see Table 1 in [29]). Local variables included annual species richness, habitat diversity, pedestrian traffic, habitat type (park/cemetery), and area (ha). Annual species richness may be positively related to the seasonal change of bird composition [12], and was measured as the accumulated number of species registered in each site during both seasons in each site (mean = 21.08 species, range = 5–56). Habitat diversity is probably positively associated with the seasonal persistence of bird species [5] and was estimated by calculating the Shannon index on the percent cover of the seven habitat cover types including the percent cover of built area, tree, lawn, shrub, non-managed herbaceous vegetation, bare soil, and water (mean = 1.29%, range = 0.30–1.78). The percent cover of habitat components was estimated visually in each point count. This approach is better than the use of satellite-classified images, which do not allow quantifying the vertical stratification of vegetation. In the sites with more than one point count, the variable values were averaged for each park and cemetery. Pedestrian traffic is thought to negatively affect the seasonal change of bird composition [39] and was measured as the number of pedestrians walking or standing at each point count simultaneously with bird surveys (pedestrians/10 min) (mean = 15.18 pedestrians, range = 0.5–175.75). In sites with more than one point, values of pedestrian traffic were averaged between points. Cemeteries had more built cover and less pedestrian traffic than parks (Supplementary Information, Figure S1). The area (ha) of each site was measured with the polygon function of Google Earth Pro (mean = 11.48 ha, range = 0.33–97.60).

Landscape variables were the urbanization level around each site and the population size of each city. The increasing urbanization surrounding green areas and population size of cities are thought to dampen the seasonal change of species composition [33,37]. We classified the urbanization level as “urban”, “suburban”, and “periurban” depending on the impervious cover of each site and its location in the city (see Leveau [29]). The urbanization level was characterized with Google Earth Pro images by measuring impervious cover in four plots of 9 ha located in the cardinal points. Then, the four values of the impervious surface were averaged for each site. Urban landscapes had >50% impervious cover, suburban landscapes had <50% impervious cover, and periurban landscapes were on the city fringe. We used the most recent census information from Wikipedia as our population size variable.

Large-scale variables were altitude (mean = 1073.78 m.a.s.l., range = 10–3625), annual temperature range (TRANGE), annual precipitation range (PRANGE), and hemisphere. Altitude, TRANGE, and PRANGE were obtained from Wikipedia, where information on local weather stations was provided. TRANGE and PRANGE (mm) were calculated as the difference between the lowest and the maximum monthly mean values throughout the year (mean = 9.37 °C, range = 1.1–19.3; mean = 131.25 mm, range = 18–348.8, respectively; see Leveau [37]). When data were not available in Wikipedia, we obtained the climatic values from the website climate-data.org. The hemisphere was divided into North and South.

### 2.3. The Seasonal Change of Bird Composition

The seasonal change of bird composition between breeding and non-breeding seasons was calculated using the betapart package in R version 3.6.1 [44,45]. A matrix of bird species abundance as columns and sites as rows was used to calculate the Bray–Curtis dissimilarity and its additive components of abundance gradient and balanced variation for each site with the beta.pair.abund function. Bird abundance was calculated as the maximum number of individuals detected in the two visits during each season.

Species detectability may be imperfect, particularly in assemblages with high species richness [46]. Therefore, we used a dissimilarity index that considers unseen species between seasons. Chao et al. [46] provided a Sørensen index based on species abundances that includes the effect of unseen shared species between seasons. The function chaodist of the vegan package was used [45,47].

### 2.4. Statistical Analysis

Generalized linear models (GLMs) with a Gaussian distribution of errors were used to relate the Bray–Curtis index and its two components abundance gradient and balanced variation with the 11 predictor variables in R [44]. Although sites were nested within cities, an analysis of the spatial autocorrelation of residuals using the Moran index in SAM software version 4.0 [48] revealed no significant spatial autocorrelation of residuals (*p* > 0.05). Models were obtained by backward elimination of non-significant variables (*p* > 0.05) from the full model using the ANOVA function. A Likelihood Ratio test (LRT test) was used to compare final models with null models (*p* < 0.05). R^2^ of models were obtained using the function rsq of the rsq package [49]. The multicollinearity between predictors was analyzed with car package’s vif function [50]. The generalized variance inflation index (GVIF) that handles continuous and categorical variables was used and no important collinearity between predictors was found. Model residuals were checked for heteroscedasticity, but no important patterns of residuals were found. Final models were plotted using the visreg package [51].

## 3. Results

A total of 281 species and 17,978 individuals were observed (see Supporting Information, Table S2). The Feral Pigeon (*Columba livia*), the Eared Dove (*Zenaida auriculata*), and the Monk Parakeet (*Myiopsitta monachus*) were the most abundant species.

**Figure 3:**
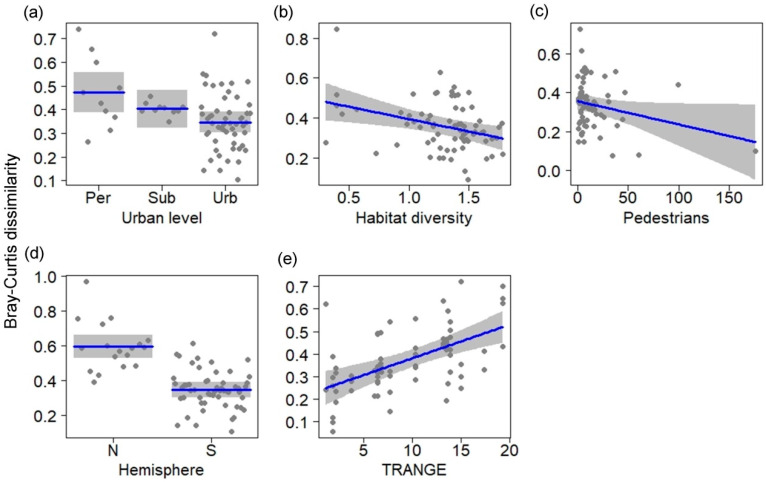
Relationship between environmental variables (**a**–**e**) and the seasonal change of bird composition (Bray–Curtis dissimilarity index) in urban parks and cemeteries of Neotropical cities. Blue lines are fitted models and grey bands are 95% confidence intervals. TRANGE: annual range of temperature (°C); Pedestrian: people/10 min; Habitat diversity: Shannon index (H′).

The Bray–Curtis seasonal composition dissimilarity was related to TRANGE, hemisphere, urban level, pedestrian traffic, and habitat diversity (Table 1; LRT = 0.98, *p* < 0.001; r^2^ = 0.46). The seasonal change of composition was the highest in sites located in peri-urban areas (Figure 3a) and decreased in sites with higher pedestrian traffic and habitat diversity (Figure 3b,c). As expected, the seasonal change of composition was the highest in the Northern Hemisphere (Figure 3d) and increased with increasing TRANGE (Figure 3e).

**Figure 4:**
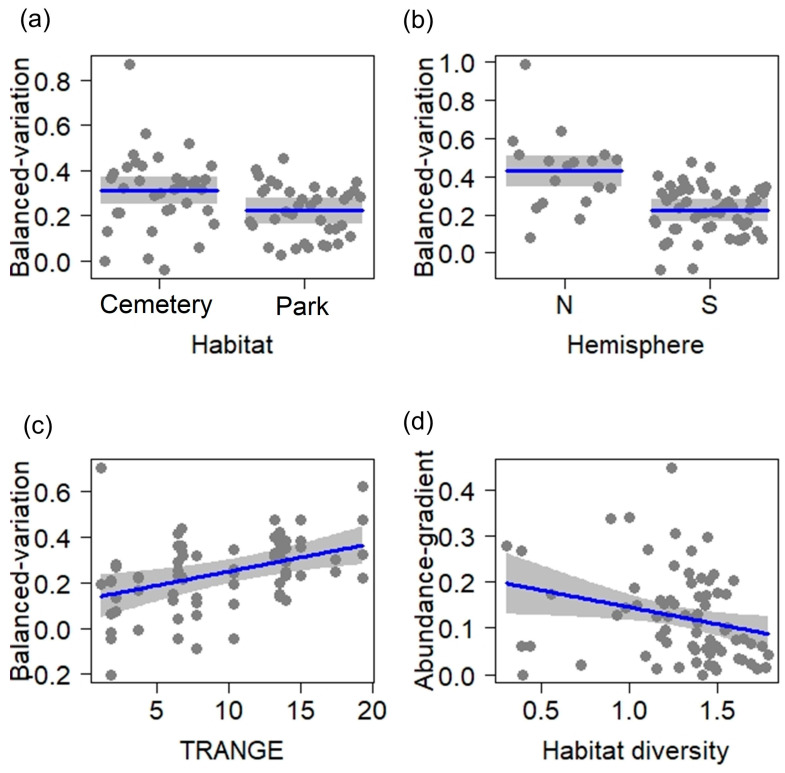
Relationship between environmental variables and (**a**–**c**) the seasonally balanced variation dissimilarity, and (**d**) the abundance gradient dissimilarity in urban parks and cemeteries of Neotropical cities. Blue lines are fitted models and grey bands are 95% confidence intervals. TRANGE: annual range of temperature (°C). Habitat diversity: Shannon index (H′).

The balanced variation dissimilarity was more important than the abundance gradient dissimilarity (mean balanced variation = 0.33 vs. mean abundance gradient = 0.13), and it was related to TRANGE, hemisphere, and habitat type (Table 1; LRT = 0.56, *p* < 0.001; r^2^ = 0.24). Therefore, the seasonal change of composition was dominated by the turnover of individuals between species. This turnover was higher in cemeteries than in parks (Figure 4a), augmented in the Northern Hemisphere (Figure 4b), and was positively related to TRANGE (Figure 4c). On the other hand, the abundance-gradient dissimilarity decreased with increasing habitat diversity (Table 1; LRT = 0.05, *p* = 0.023; r^2^ = 0.05; Figure 4d). Therefore, sites with lower habitat diversity had the highest difference in bird individuals between seasons.

**Figure 5:**
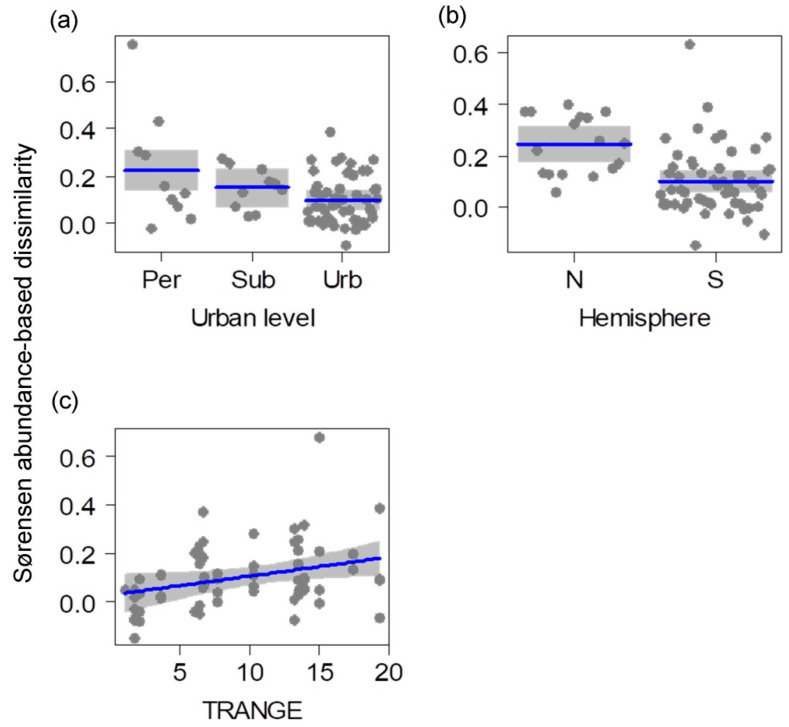
Relationship between environmental variables (**a**–**c**) and the seasonal Sørensen abundance-based dissimilarity in urban parks and cemeteries of Neotropical cities. The Sørensen index considers unseen species between seasons. Blue lines are fitted models and grey bands are 95% confidence intervals. TRANGE: annual range of temperature (°C).

The Sørensen dissimilarity abundance-based index that considers unseen species between seasons varied significantly with urbanization level, hemisphere, and annual temperature range (Table 1; LRT = 0.33, *p* < 0.001; r^2^ = 0.19; Figure 5). The seasonal change of composition decreased in the most urbanized sites and increased in the northern hemisphere part of Latin America. Moreover, the seasonal compositional change positively correlated with the annual temperature range.

## 4. Discussion

**Figure 6:**
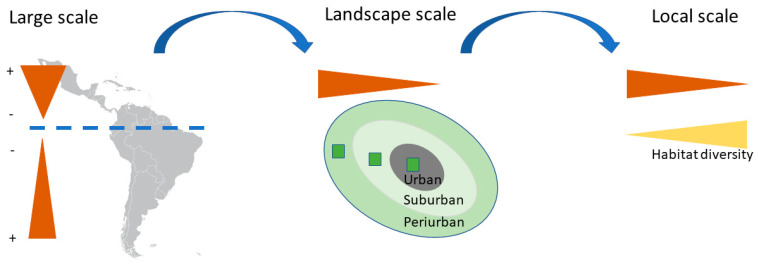
Summary of the results found in our study. A multiscale framework shows the different drivers of bird assemblage seasonality in the Neotropics. The orange triangles represent the amount of seasonal change of species composition. At the large scale, the blue dashed line indicates the Equator. The seasonal change of bird composition increases with increasing annual range of temperature, which is positively related to latitude (r = 0.91, *p* < 0.05). Moreover, the seasonal change in bird composition also increases in the Northern Hemisphere part of the Neotropics. At the landscape scale, the seasonal change is negatively related to urbanization (scales from grey to green), whereas at the local scale is negatively related to habitat diversity (yellow triangle).

Our results showed that variables at different spatial scales were related to the seasonal changes in bird composition of urban green areas in the Neotropics (Figure 6). As expected, a large-scale variable such as the seasonal change of temperature was positively related to bird composition seasonality. These results are congruent with previous studies that suggest an important effect of temperature on the distribution and movements of avian species in natural communities [52,53,54,55]. Moreover, our results indicated that the sites located in the Northern Hemisphere section of the Neotropics showed a higher seasonality of bird composition than equal latitudes in the Southern Hemisphere. At the landscape scale urbanization level was related to bird composition seasonality. At a local scale, habitat diversity was positively associated with the annual stability of bird composition. The results obtained also revealed that different environmental variables were related to the two components of seasonal compositional change, a nestedness pattern of individuals (abundance gradient) and a turnover pattern of individuals (balanced variation).

The annual species richness was not related to the seasonal change of species composition, suggesting that there was no sampling effect in our patterns [12]. Thus, our findings suggest that the seasonal beta diversity is driven by ecological mechanisms such as climate changes and habitat associations.

On the other hand, the use of the Sørensen index that considered unseen species between seasons showed similar patterns to the Bray–Curtis index with the environmental variables. Therefore, the impacts of annual temperature range, urbanization level, and hemisphere on the seasonal species dissimilarity were not obscured by sampling effects between seasons.

The seasonal changes of composition were positively related to the seasonal temperature change, and this result agrees with others obtained in Europe and North America [8,9,12]. The seasonal change in temperature is related to changes in food resources for birds, such as plant material, insects, nectar, and fruit [6]. The compositional seasonality was also higher in sites located in the Northern Hemisphere. This pattern agrees with our hypothesis that sites located in the northern fringe of the Neotropical region, such as Mexico and Costa Rica that receive an influx of Nearctic migrants in autumn–winter, have a greater seasonal change of bird composition than sites located in South America.

The increased urbanization surrounding each site decreased the bird composition seasonality, supporting the results obtained by other authors worldwide [17,32,39,56]. This reduction of seasonality in the most urbanized areas could be related to two main factors: (1) the increased dominance of omnivorous and resident species that can take advantage of the food resources provided by humans year-round [33,39,56]; and (2) the decreased presence and abundance of migratory species, which have lower food resources in the most urbanized areas and are more affected by human disturbance [37,57,58].

At the local scale, sites with increased habitat diversity had more annual stability of bird composition. On the one hand, this result agrees with the patterns found by Karr [5], who postulated that habitat diversity buffered the seasonal changes of food resources. In urban green areas, sites with high habitat diversity are usually composed of several exotic tree species that provide food resources throughout the year, unlike native tree species with a marked productive season of food resources [33]. On the other hand, our results could be related to the habitat preferences of migrant species, which prefer the dominance of a particular habitat type instead of a diversity of habitats. For example, several North American migrants wintering in Mexico City, such as Yellow-rumped Warbler (*Setophaga coronata*) and the Wilson’s warbler (*Cardellina pusilla*), have been more frequent in sites with increased tree cover [59]. Similarly, several South American migrants that summer in Central Argentina, such as the Brown-chested Martin (*Progne tapera*) and the White-rumped Swallow (*Tachycineta leucorrhoa*), prefer open areas [60,61,62].

Pedestrian traffic was negatively related to the seasonal composition dissimilarity. Therefore, sites with the highest pedestrian traffic had a more stable annual bird species composition. Pedestrians may supply food resources, such as pieces of bread, to generalist species, thus allowing these species to be present year-round. On the other hand, pedestrians may disturb and exclude migratory birds, thus lowering the seasonal dynamics of species composition.

The decomposition of the Bray–Curtis dissimilarity index in their additive components showed that balanced variation had a higher proportion of the total composition dissimilarity than abundance gradient, indicating a predominant turnover of individuals of different species between seasons. This turnover increased in areas with higher temperature seasonality and in the Northern Hemisphere. At least two processes could be driving the patterns observed. Firstly, the arrival of latitudinal migrants to a site is associated with some species’ departure, which makes longitudinal or altitudinal movements [63,64,65]. Secondly, resident species have behavioral changes throughout the year, forming flocks during winter and concentrating in particular areas [65,66,67]. Therefore, the concentration of species in particular sites and their absence in other sites where the winter migrants are present may result in an annual turnover of individuals.

The balanced variation was higher in cemeteries than in parks. Therefore, cemeteries had a higher turnover of individuals of different species between breeding and non-breeding seasons than parks. Parks have more pedestrian traffic than cemeteries, and pedestrian traffic was shown to affect negatively the presence of migrants [57,68]. Thus, a lower proportion of migrants can decrease the seasonality of bird communities in urban parks. On the other hand, higher pedestrian traffic in parks could be related to more food provided by humans to resident omnivorous species, which stabilizes the seasonal change of bird composition.

The abundance gradient decreased with increasing habitat diversity, suggesting that sites with less habitat diversity or dominated by a habitat component increased the individual loss or gains between seasons. Sites dominated by a habitat component, such as trees or grass, are probably visited by migrants but are scarcely used by residents year-round. On the other hand, due to migrants using the food surplus not consumed by residents in a given habitat type [9,10], the greater the proportion of the vegetation layer may result in a greater amount of food surplus, and thus of migrants.

This study could be improved by more accurate habitat diversity measurements. For example, the use of remote sensors such as LIDAR (Light Detection and Ranging) may be useful to measure the 3D structure of urban habitats [69]. On the other hand, this study compared bird compositional seasonality of two types of urban green areas. More insights about the effect of urbanization on the seasonality of bird communities could be obtained by comparing urban habitats with natural habitats.

## 5. Conclusions

Our results revealed that a multiscale framework is necessary to understand the seasonal change of bird communities facing the actual global change crisis (Figure 6). Climatic variables along the year were the main drivers of the seasonal change in urban species composition in the Neotropics. Additionally, the larger seasonal changes of bird communities were found in the North Hemisphere section of the Neotropics, possibly because these communities are receiving a significant influx of Nearctic migrants. Our analysis revealed that local and landscape variables, such as habitat diversity and urbanization surrounding each park and cemetery, drove the seasonality of bird composition.

The partitioning of composition seasonality revealed that the two components, balanced variation and abundance gradient, were related to different environmental drivers. The dominant pattern was balanced variation dissimilarity, showing that in the Neotropics the turnover of individuals of different species between breeding and non-breeding seasons was the predominant driver of assemblage seasonality in urban areas. On the other hand, habitat diversity was negatively related to the abundance gradient dissimilarity, suggesting that sites with several habitat components stabilized bird composition’s seasonal change.

The results obtained highlight the importance of considering not only the spatial diversity of birds but also the temporal component of diversity. Therefore, the conservation and restoration of green areas in cities of the Neotropical Region must consider the seasonal changes of bird communities, as well as their geographical location and local-scale habitat features.

## Figures and Tables

**Table 1:** Results of the best final generalized linear models showing the relationship between the environmental variables and (a) seasonal change in bird composition (Bray–Curtis dissimilarity), (b) seasonal balanced abundance dissimilarity, (c) seasonal abundance gradient dissimilarity, and (d) the Sørensen abundance-based dissimilarity. Level Periurban, Northern Hemisphere, and Habitat cemetery are in the intercept. TRANGE: annual range of temperature.

	Estimate	SE	t Value	*p*
(a) Bray–Curtis dissimilarity				
Intercept	0.786	0.079	9.901	<0.001
Level—Suburban	−0.069	0.058	−1.179	0.243
Level—Urban	−0.126	0.045	−2.811	0.006
Habitat diversity (H′)	−0.124	0.043	−2.887	0.005
Pedestrians	−0.001	0.001	−1.990	0.051
Hemisphere—South	−0.248	0.040	−6.127	<0.001
TRANGE	0.015	0.003	4.310	<0.001
(b) Balanced variation dissimilarity				
Intercept	0.423	0.043	9.877	<0.001
Habitat—Park	−0.088	0.035	−2.536	0.013
Hemisphere—South	−0.208	0.049	−4.249	<0.001
TRANGE	0.012	0.004	3.036	0.003
(c) Abundance gradient dissimilarity				
Intercept	0.219	0.043	5.051	<0.001
Habitat diversity (H′)	−0.074	0.033	−2.267	0.027
(d) Sørensen abundance-based dissimilarity				
Intercept	0.300	0.062	4.846	<0.001
Level—Suburban	−0.073	0.062	−1.189	0.239
Level—Urban	−0.126	0.046	−2.712	0.009
Hemisphere—South	−0.145	0.042	−3.428	0.001
TRANGE	0.008	0.004	2.125	0.037

## Data Availability

The datasets generated and/or analyzed during the current study are available upon request to the corresponding author.

## Supplementary Materials

The following supporting information can be downloaded at: https://www.mdpi.com/article/10.3390/ani14243564/s1, Table S1: Environmental information of the cities included in the analysis and with the amount of parks and cemeteries surveyed in each city; Table S2: List of species recorded in urban parks and cemeteries of Latin America during breeding and non-breeding seasons. Numbers are the mean abundance per site, standard deviation and number of sites where species were recorded. Figure S1: Boxplots showing the two variables that best discriminated between parks and cemeteries in urban areas of the Neotropics, based of Discriminant function analysis (Correctness rate = 0.8). (a) Percent cover of Built, and (b) Pedestrian/10 min.
