# Supplementary material for: Drivers of Seasonal Change of Avian Communities in Urban Parks and Cemeteries of Latin America
Source: Animals (Basel). 2024 Dec 10;14(24):3564. doi: 10.3390/ani14243564 (PMC11672830; doi:10.3390/ani14243564)
Supplement: Supplementary file 1 [file animals-14-03564-s001.zip › animals-3290145-supplementary.pdf]

Figure S1. Boxplots showing the two variables that best discriminated between parks and cemeteries in urban areas of the Neotropics, based of Discriminant function analysis (Correctness rate = 0.8). a) Percent cover of Built, and b) Pedestrian/ 10 minutes.

a)

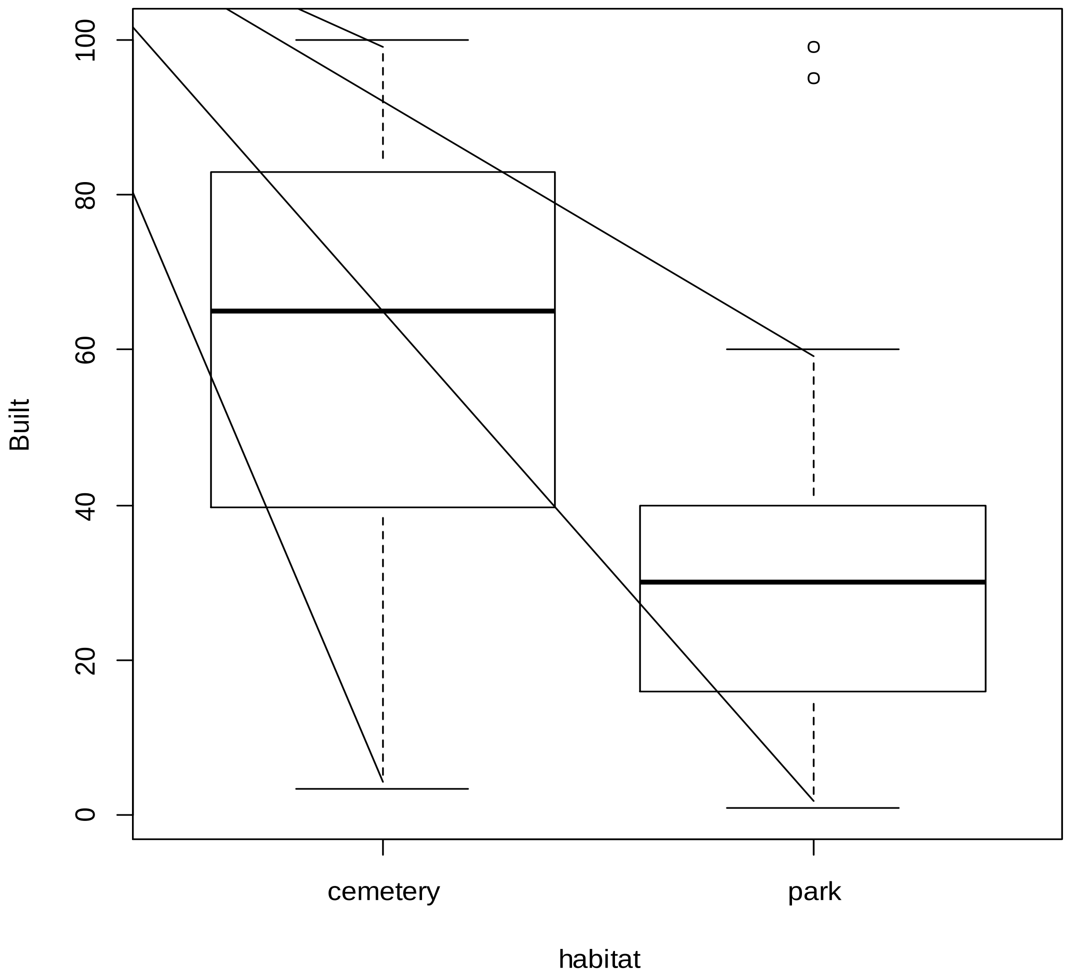

b)

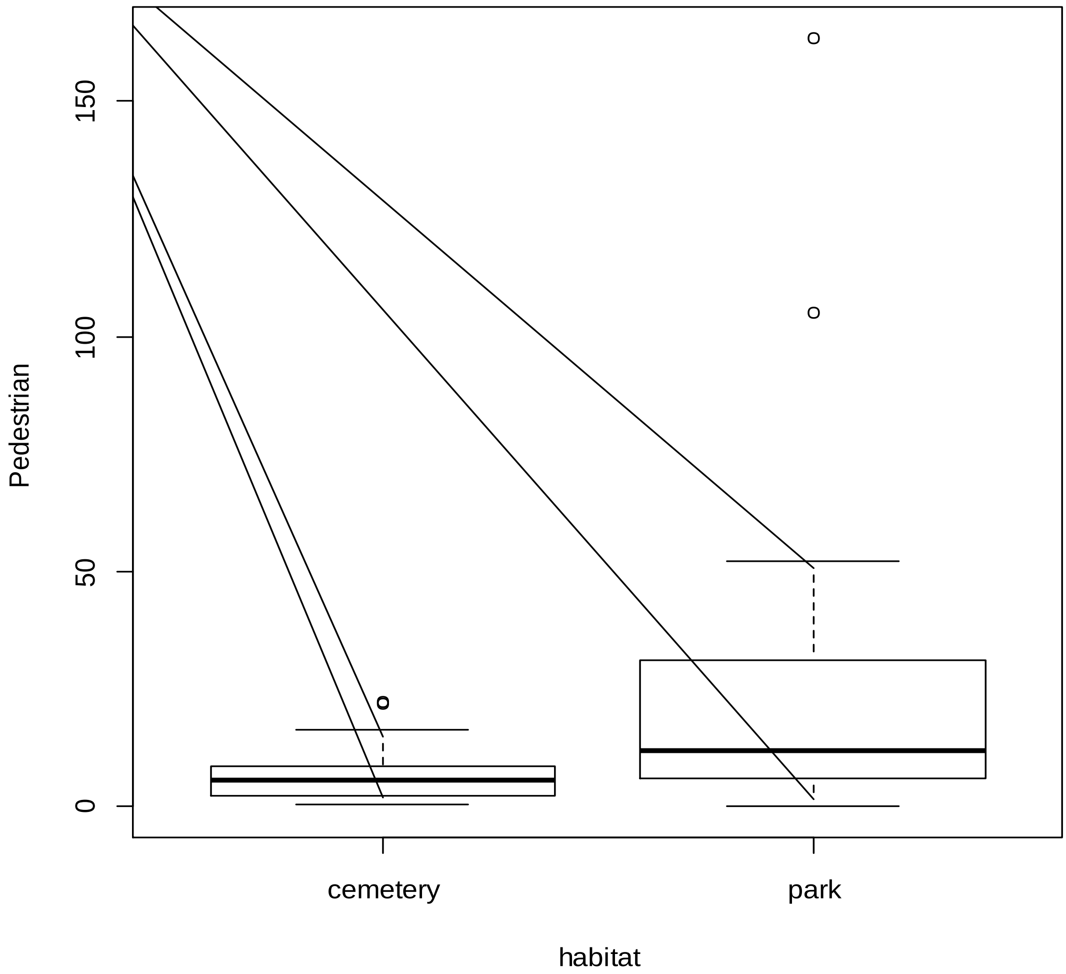

Table S1. Environmental information of the cities included in the analysis and with the amount of parks and cemeteries surveyed in each city.

City	Country	Coordinates	Altitude (masl)	Population	Parks	Cemeteries
Buenos Aires	Argentina	34°35′59″S 58°22′55″W	25	17541141	3	3
Chilecito	Argentina	29°10′00″S 67°30′00″W	1080	41179	2	2
Mendoza	Argentina	32°53′00″S 68°50′00″W	769	1400000	1	1
San Juan	Argentina	31°32′15″S 68°32′11″W	640	471389	2	2
San Miguel de Tucumán/ Yerba Buena	Argentina	26°49′00″S 65°13′00″W	431	1030526	2	2
Santa Fe	Argentina	31°38′00″S 60°42′00″W	25	490171	1	1
La Paz	Bolivia	16°29′39″S 68°08′51″W	3625	1801539	2	2
Curitiba	Brazil	25°25′47″S 49°16′19″W	934	1864416	3	2

Porto Alegre	Brazil	30°01′58″S 51°13′48″W	10	1409939	2	2
Santa María	Brazil	29°41′02″S 53°48′25″W	113	261980	1	1
Sorocaba	Brazil	23°30′07″S 47°27′28″W	601	644919	1	1
Taubaté	Brazil	23°02′00″S 45°33′00″W	589	273426	2	2
Santiago de Chile	Chile	33°27′00″S 70°40′00″W	520	6257516	4	4
Medellín	Colombia	6°14′41″N 75°34′29″W	1495	2533424	1	1
San José	Costa Rica	9°56′00″N 84°05′00″W	1300	2723850	3	3
Ciudad de México	Mexico	19°25′10″N 99°08′44″W	2240	22597699	4	4
Xalapa-Enriquez	Mexico	19°31′52″N 96°54′57″W	1420	424755	1	1
Arequipa	Peru	16°23′56″S 71°32′13″W	2335	1117284	2	2

Table S2. List of species recorded in urban parks and cemeteries of Latin America during breeding and non-breeding seasons. Numbers are the mean abundance per site, standard deviation and number of sites where species were recorded.

	Park						Cemetery					
	Breeding			Non-breeding			Breeding			Non-breeding		
Species	Mean	SD	N	Mean	SD	N	Mean	SD	N	Mean	SD	N
Anhinga_anhinga	0.05	0.33	1	0.00	0.00	0	0.00	0.00	0	0.00	0.00	0
Phalacrocorax_brasilianus	0.51	2.96	2	0.70	4.11	2	0.00	0.00	0	0.00	0.00	0
Ardea_cocoi	0.00	0.00	0	0.03	0.16	1	0.00	0.00	0	0.03	0.17	1
Ardea_alba	0.22	0.67	4	0.22	0.71	4	0.06	0.23	2	0.03	0.17	1
Egretta_thula	0.22	0.95	2	0.11	0.39	3	0.03	0.17	1	0.06	0.33	1
Bubulcus_ibis	0.38	2.30	1	0.22	1.32	1	0.03	0.17	1	0.00	0.00	0
Syrigma_sibilatrix	0.05	0.33	1	0.03	0.16	1	0.00	0.00	0	0.00	0.00	0
Butorides_striatus	0.03	0.16	1	0.00	0.00	0	0.00	0.00	0	0.00	0.00	0
Nycticorax_nycticorax	0.41	1.86	3	0.08	0.36	2	0.17	1.00	1	0.06	0.33	1
Phimosus_infuscatus	0.14	0.67	2	0.00	0.00	0	0.00	0.00	0	0.00	0.00	0
Theristicus_caudatus	0.05	0.33	1	0.00	0.00	0	0.17	1.00	1	0.11	0.67	1
Mesembrenibis_cayanensis	0.11	0.66	1	0.00	0.00	0	0.00	0.00	0	0.00	0.00	0
Coragyps_atratus	0.08	0.36	2	0.11	0.39	3	0.11	0.40	3	0.11	0.46	2
Amazonetta_brasiliensis	0.22	1.32	1	0.11	0.66	1	0.06	0.33	1	0.00	0.00	0
Geranoaetus_polyosoma	0.03	0.16	1	0.03	0.16	1	0.00	0.00	0	0.00	0.00	0
Rupornis_magnirostris	0.11	0.39	3	0.00	0.00	0	0.08	0.37	2	0.06	0.23	2
Parabuteo_unicinctus	0.08	0.49	1	0.05	0.23	2	0.08	0.37	2	0.22	1.02	3
Rosthramus_sociabilis	0.08	0.49	1	0.00	0.00	0	0.00	0.00	0	0.00	0.00	0
Accipiter_sp	0.00	0.00	0	0.00	0.00	0	0.08	0.50	1	0.00	0.00	0
Caracara_plancus	0.27	0.73	6	0.32	0.82	7	0.28	0.78	6	0.06	0.33	1
Phalcoboenus_chimango	0.08	0.36	2	0.24	0.95	3	0.75	1.44	11	0.50	2.01	3
Milvago_chimachima	0.00	0.00	0	0.03	0.16	1	0.00	0.00	0	0.00	0.00	0
Falco_rufigularis	0.03	0.16	1	0.00	0.00	0	0.00	0.00	0	0.00	0.00	0
Falco_sparverius	0.11	0.52	2	0.05	0.23	2	0.22	0.54	6	0.39	0.90	7
Penelope_obscura	0.08	0.36	2	0.00	0.00	0	0.00	0.00	0	0.00	0.00	0

Callipepla__californica	0.00	0.00	0	0.00	0.00	0	0.11	0.46	2	0.22	0.96	2
Aramides_saracura	0.27	0.80	4	0.14	0.48	3	0.06	0.23	2	0.06	0.23	2
Pardirallus_nigricans	0.03	0.16	1	0.03	0.16	1	0.00	0.00	0	0.00	0.00	0
Gallinula_galeata	0.54	2.58	2	0.62	3.62	2	0.00	0.00	0	0.00	0.00	0
Aramus_guarauna	0.03	0.16	1	0.00	0.00	0	0.00	0.00	0	0.00	0.00	0
Jacana_jacana	0.00	0.00	0	0.08	0.49	1	0.00	0.00	0	0.00	0.00	0
Vanellus_chilensis	1.41	4.21	6	1.43	5.10	7	2.31	5.44	11	2.75	5.84	10
Vanellus_resplendens	0.00	0.00	0	0.00	0.00	0	0.00	0.00	0	0.64	3.83	1
Sterna_supercilliaris	0.00	0.00	0	0.03	0.16	1	0.00	0.00	0	0.00	0.00	0
Columba_livia	21.00	40.82	25	20.46	37.04	24	14.69	32.29	17	21.22	39.20	18
Patagioenas_flavirostris	0.65	2.76	2	0.14	0.48	3	0.06	0.23	2	0.28	1.11	3
Patagioenas_maculosa	3.49	7.64	9	6.81	18.73	8	1.06	3.30	7	1.56	3.71	8
Patagioenas_picazuro	3.19	7.71	10	2.62	5.96	10	2.64	8.08	9	1.89	5.67	6
Streptopelia_decaocto	0.00	0.00	0	0.00	0.00	0	0.08	0.50	1	0.00	0.00	0
Zenaida_asiatica	0.49	1.80	3	0.54	1.86	3	0.19	0.71	3	0.14	0.68	2
Zenaida_auriculata	16.38	26.17	22	6.24	9.89	22	8.97	13.90	19	6.33	14.40	19
Zenaida_macroura	0.00	0.00	0	0.00	0.00	0	0.03	0.17	1	0.00	0.00	0
Zenaida_meloda	0.73	3.11	2	0.81	3.47	2	0.36	1.85	2	0.92	3.88	2
Columbina_cruziana	0.65	2.99	2	0.95	5.43	2	1.03	5.37	2	1.44	6.91	2
Columbina_inca	1.24	6.13	3	1.30	7.89	1	0.00	0.00	0	0.22	0.87	4
Columbina_picui	1.35	3.61	9	0.54	1.80	5	0.44	1.18	6	0.42	1.36	5
Columbina_talpacoti	0.97	3.10	6	0.73	2.48	6	1.08	3.98	4	0.58	1.56	6
Metropelia_ceciliae	0.00	0.00	0	0.05	0.33	1	0.92	4.53	4	1.28	5.43	2
Leptotila_verreauxi	0.14	0.67	2	0.00	0.00	0	0.17	1.00	1	0.06	0.23	2
Leptotila_rufaxila	0.00	0.00	0	0.03	0.16	1	0.06	0.23	2	0.03	0.17	1
Psittacara_finschi	0.05	0.33	1	0.35	1.21	3	0.00	0.00	0	0.00	0.00	0
Psittacara_leucophthalmus	0.95	2.70	5	0.97	3.23	5	1.33	4.01	5	0.53	2.46	2
Thectocercus_acuticaudatus	0.00	0.00	0	0.14	0.82	1	0.00	0.00	0	2.39	13.34	2
Aratinga_nenday	0.14	0.82	1	0.00	0.00	0	1.03	6.17	1	0.92	5.50	1
Aratinga_mitratus	0.22	1.32	1	0.00	0.00	0	0.08	0.50	1	0.03	0.17	1
Myopsitta_monacha	4.62	12.52	9	8.30	20.37	11	4.06	9.94	11	9.17	20.41	11
Pyrrhura_frontalis	0.00	0.00	0	0.14	0.82	1	0.06	0.33	1	0.00	0.00	0
Pionus_maximilianus	0.00	0.00	0	0.03	0.16	1	0.00	0.00	0	0.00	0.00	0
Forpus_xanthopterygius	0.00	0.00	0	0.43	2.06	2	0.00	0.00	0	0.00	0.00	0
Brotogeris_chiriri	1.49	5.21	5	0.32	1.03	4	0.11	0.46	2	0.28	0.97	3
Brotogeris_tirica	0.19	0.84	3	0.11	0.52	2	0.39	1.34	3	0.31	1.26	3
Amazona_aestiva	0.08	0.49	1	0.24	0.95	3	0.00	0.00	0	0.06	0.33	1
Amazona_albifrons	0.24	1.48	1	0.00	0.00	0	0.06	0.33	1	0.06	0.33	1
Melopsittacus_undulatus	0.00	0.00	0	0.11	0.66	1	0.00	0.00	0	0.00	0.00	0
Guira_guira	0.38	1.23	6	0.11	0.52	2	0.28	1.03	5	0.33	1.20	4
Crotophaga_ani	0.03	0.16	1	0.11	0.66	1	0.17	0.70	3	0.00	0.00	0
Piaya_cayana	0.08	0.28	3	0.08	0.36	2	0.03	0.17	1	0.03	0.17	1
Athene_cunicularia	0.03	0.16	1	0.00	0.00	0	0.08	0.37	2	0.22	0.93	2
Asio_stigius	0.00	0.00	0	0.00	0.00	0	0.00	0.00	0	0.03	0.17	1
Streptoprocne_zonaris	0.00	0.00	0	0.00	0.00	0	0.17	0.85	2	0.03	0.17	1
Chaetura_meridionalis	0.08	0.49	1	0.00	0.00	0	0.25	1.50	1	0.00	0.00	0

Chaetura_vauxi	0.19	1.15	1	0.00	0.00	0	0.11	0.67	1	0.00	0.00	0
Colibri_coruscans	0.14	0.67	2	0.35	1.53	2	0.17	0.70	2	0.28	1.19	2
Colibri_serrirostris	0.03	0.16	1	0.00	0.00	0	0.14	0.68	2	0.03	0.17	1
Leucochloris_albicollis	0.00	0.00	0	0.03	0.16	1	0.11	0.40	3	0.00	0.00	0
Amazilia_beryllina	0.19	0.81	2	0.16	0.73	2	0.25	1.05	3	0.25	0.84	3
Amazilia_chionogaster	0.00	0.00	0	0.00	0.00	0	0.03	0.17	1	0.03	0.17	1
Amazilia_cyanocephala	0.00	0.00	0	0.03	0.16	1	0.06	0.33	1	0.08	0.50	1
Amazilia_tzacatl	0.05	0.33	1	0.08	0.28	3	0.06	0.33	1	0.17	0.70	2
Thalurania_glaucopis	0.03	0.16	1	0.05	0.33	1	0.03	0.17	1	0.00	0.00	0
Eupetomena_macroura	0.24	0.93	3	0.32	1.81	2	0.08	0.37	2	0.08	0.28	3
Sephanoides__sephaniodes	0.00	0.00	0	1.14	4.45	4	0.00	0.00	0	0.94	3.83	4
Chlorostilbon_lucidus	0.27	0.77	6	0.16	0.44	5	0.36	1.02	6	0.06	0.23	2
Pampa_curvipennis	0.00	0.00	0	0.00	0.00	0	0.03	0.17	1	0.00	0.00	0
Hylocharis_chrysura	0.11	0.66	1	0.14	0.48	3	0.00	0.00	0	0.22	0.76	3
Patagona_gigas	0.00	0.00	0	0.03	0.16	1	0.00	0.00	0	0.00	0.00	0
Thaumastura_cora	0.00	0.00	0	0.05	0.23	2	0.00	0.00	0	0.25	1.50	1
Rodhopis_vesper	0.27	1.24	2	0.14	0.59	2	0.31	1.28	2	0.08	0.50	1
Myrtis_fanny	0.08	0.49	1	0.00	0.00	0	0.00	0.00	0	0.03	0.17	1
Sappho_sparganurus	0.00	0.00	0	0.11	0.52	2	0.00	0.00	0	0.03	0.17	1
Cynanthus_latirostris	0.05	0.23	2	0.03	0.16	1	0.19	0.67	3	0.03	0.17	1
Trogon_surrucura	0.00	0.00	0	0.03	0.16	1	0.00	0.00	0	0.00	0.00	0
Chloroceryle_americana	0.05	0.23	2	0.00	0.00	0	0.00	0.00	0	0.00	0.00	0
Megaceryle_torquata	0.00	0.00	0	0.00	0.00	0	0.03	0.17	1	0.00	0.00	0
Colaptes_campestris	0.14	0.67	2	0.05	0.33	1	0.17	0.61	3	0.22	0.93	2
Colaptes_melanocholoros	0.27	0.69	6	0.14	0.54	3	0.14	0.49	3	0.19	0.58	5
Colaptes_rupicola	0.22	1.32	1	0.00	0.00	0	0.14	0.83	1	0.00	0.00	0
Melanerpes_aurifrons	0.08	0.49	1	0.08	0.49	1	0.08	0.50	1	0.03	0.17	1
Melanerpes_cactorum	0.05	0.33	1	0.03	0.16	1	0.00	0.00	0	0.00	0.00	0
Melanerpes_candidus	0.05	0.23	2	0.00	0.00	0	0.06	0.23	2	0.11	0.67	1
Melanerpes_hoffmannii	0.11	0.66	1	0.24	1.19	2	0.00	0.00	0	0.03	0.17	1
Dryobates_mixtus	0.08	0.49	1	0.11	0.52	2	0.00	0.00	0	0.00	0.00	0
Dryobates_scalaris	0.00	0.00	0	0.05	0.33	1	0.06	0.33	1	0.06	0.33	1
Picumnus_cirratus	0.03	0.16	1	0.05	0.23	2	0.06	0.33	1	0.00	0.00	0
Picumnus_temminckii	0.46	2.47	3	0.00	0.00	0	0.00	0.00	0	0.11	0.67	1
Sphyrapicus_varius	0.00	0.00	0	0.05	0.33	1	0.00	0.00	0	0.00	0.00	0
Campephilus_melanoleucos	0.00	0.00	0	0.03	0.16	1	0.00	0.00	0	0.00	0.00	0
Cariama_cristata	0.00	0.00	0	0.03	0.16	1	0.00	0.00	0	0.00	0.00	0
Furnarius_rufus	5.14	7.63	19	6.70	10.93	20	4.28	7.59	15	4.19	8.53	15
Certhiaxis_cinnamomeus	0.03	0.16	1	0.05	0.33	1	0.00	0.00	0	0.00	0.00	0
Cranioleuca_pallida	0.00	0.00	0	0.03	0.16	1	0.00	0.00	0	0.00	0.00	0
Cranioleuca_pyrrhophia	0.03	0.16	1	0.03	0.16	1	0.00	0.00	0	0.00	0.00	0
Leptasthenura__aegithaloides	0.00	0.00	0	0.00	0.00	0	0.03	0.17	1	0.11	0.52	2
Leptasthenura_setaria	0.30	1.08	3	0.22	0.89	3	0.00	0.00	0	0.00	0.00	0
Pseudoseisura_lophotes	0.05	0.23	2	0.14	0.67	2	0.03	0.17	1	0.19	0.67	3
Xiphorhynchus_fuscus	0.03	0.16	1	0.00	0.00	0	0.00	0.00	0	0.00	0.00	0
Lepidocolaptes_angustirostris	0.30	1.22	3	0.24	1.04	2	0.11	0.46	2	0.17	0.85	2

Chiroxiphia_caudata	0.05	0.33	1	0.05	0.33	1	0.00	0.00	0	0.00	0.00	0
Thamnophilus_caerulecens	0.00	0.00	0	0.03	0.16	1	0.00	0.00	0	0.00	0.00	0
Thamnophilus_doliatus	0.00	0.00	0	0.05	0.33	1	0.00	0.00	0	0.00	0.00	0
Dysithamnus_mentalis	0.08	0.28	3	0.08	0.28	3	0.00	0.00	0	0.00	0.00	0
Conopophaga_lineata	0.03	0.16	1	0.00	0.00	0	0.00	0.00	0	0.00	0.00	0
Todirostrum_cinereum	0.05	0.33	1	0.14	0.67	2	0.03	0.17	1	0.03	0.17	1
Camptostoma_imberbe	0.03	0.16	1	0.00	0.00	0	0.00	0.00	0	0.06	0.23	2
Camptostoma_obsoletum	0.08	0.28	3	0.00	0.00	0	0.06	0.23	2	0.14	0.59	2
Elaenia_sp.	0.00	0.00	0	0.03	0.16	1	0.00	0.00	0	0.00	0.00	0
Elaenia__albiceps	0.14	0.82	1	0.00	0.00	0	0.33	1.84	2	0.00	0.00	0
Elaenia_flavogaster	0.08	0.36	2	0.08	0.36	2	0.14	0.59	2	0.17	1.00	1
Elaenia_obscura	0.00	0.00	0	0.00	0.00	0	0.06	0.33	1	0.00	0.00	0
Elaenia_spectabilis	0.00	0.00	0	0.00	0.00	0	0.19	1.17	1	0.06	0.33	1
Phyllomyias_fasciatus	0.16	0.99	1	0.03	0.16	1	0.00	0.00	0	0.00	0.00	0
Contopus_cooperi	0.03	0.16	1	0.00	0.00	0	0.03	0.17	1	0.00	0.00	0
Contopus_pertinax	0.00	0.00	0	0.03	0.16	1	0.00	0.00	0	0.03	0.17	1
Contopus_sordidulus	0.00	0.00	0	0.00	0.00	0	0.00	0.00	0	0.03	0.17	1
Empidonax_hammondii	0.00	0.00	0	0.03	0.16	1	0.00	0.00	0	0.00	0.00	0
Empidonax_occidentalis	0.05	0.33	1	0.00	0.00	0	0.00	0.00	0	0.00	0.00	0
Suiriri_suiriri	0.00	0.00	0	0.16	0.99	1	0.00	0.00	0	0.03	0.17	1
Serpophaga_subcristata	0.16	0.50	4	0.16	0.50	4	0.28	1.00	3	0.33	1.29	3
Pyrocephalus_rubinus	0.00	0.00	0	0.00	0.00	0	0.14	0.68	2	0.00	0.00	0
Anairetes_parulus	0.11	0.52	2	0.54	1.82	5	0.17	0.70	3	0.53	1.92	3
Myiodynastes_luteiventris	0.14	0.82	1	0.00	0.00	0	0.00	0.00	0	0.00	0.00	0
Myiodinastes_maculatus	0.05	0.23	2	0.00	0.00	0	0.31	1.41	2	0.00	0.00	0
Myiodinastes_solitarius	0.03	0.16	1	0.00	0.00	0	0.00	0.00	0	0.00	0.00	0
Empidonomus_varius	0.05	0.33	1	0.00	0.00	0	0.00	0.00	0	0.00	0.00	0
Pitangus_sulphuratus	4.89	7.91	18	5.08	7.56	20	4.28	6.11	22	3.92	4.95	21
Tyrannus_melancholicus	0.65	1.75	8	0.38	0.95	6	0.83	1.70	9	0.42	0.97	7
Tyrannus_savana	0.54	2.02	6	0.00	0.00	0	0.53	2.04	6	0.06	0.33	1
Tyrannus_vociferans	0.05	0.33	1	0.03	0.16	1	0.00	0.00	0	0.03	0.17	1
Machetornis_rixosa	0.78	2.84	8	1.00	4.29	7	0.67	2.03	7	0.50	1.63	5
Megarynchus_pitangua	0.05	0.23	2	0.03	0.16	1	0.06	0.23	2	0.11	0.52	2
Myiozetetes_cayanensis	0.00	0.00	0	0.00	0.00	0	0.00	0.00	0	0.08	0.50	1
Myiozetetes_similis	0.16	0.50	4	0.24	0.93	3	0.75	3.52	4	0.64	3.03	4
Fluvicola_nengeta	0.30	1.13	3	0.27	1.19	3	0.06	0.33	1	0.08	0.50	1
Hymenops_perspicillatus	0.05	0.33	1	0.00	0.00	0	0.00	0.00	0	0.00	0.00	0
Phytotoma__rara	0.03	0.16	1	0.03	0.16	1	0.00	0.00	0	0.06	0.33	1
Phytotoma_rutila	0.14	0.48	3	0.00	0.00	0	0.03	0.17	1	0.00	0.00	0
Cyclaris_gujanensis	0.14	0.42	4	0.08	0.28	3	0.00	0.00	0	0.00	0.00	0
Vireo_olivaceus	0.00	0.00	0	0.00	0.00	0	0.03	0.17	1	0.00	0.00	0
Vireo_flavoviridis	0.00	0.00	0	0.05	0.33	1	0.00	0.00	0	0.00	0.00	0
Vireo_gilvus	0.03	0.16	1	0.00	0.00	0	0.03	0.17	1	0.00	0.00	0
Vireo_belli	0.00	0.00	0	0.00	0.00	0	0.08	0.50	1	0.00	0.00	0
Vireo_huttoni	0.00	0.00	0	0.00	0.00	0	0.03	0.17	1	0.00	0.00	0
Vireo_griseus	0.00	0.00	0	0.00	0.00	0	0.00	0.00	0	0.08	0.50	1

Vireo_plumbeus	0.00	0.00	0	0.03	0.16	1	0.00	0.00	0	0.00	0.00	0
Vireo_cassini	0.00	0.00	0	0.00	0.00	0	0.00	0.00	0	0.03	0.17	1
Cyanocorax_morio	0.05	0.33	1	0.00	0.00	0	0.42	2.50	1	0.22	1.33	1
Cyanocorax_chrysops	0.05	0.23	2	0.00	0.00	0	0.06	0.33	1	0.00	0.00	0
Progne_elegans	0.05	0.33	1	0.00	0.00	0	0.03	0.17	1	0.00	0.00	0
Progne_chalybea	0.46	1.92	3	0.00	0.00	0	0.53	2.83	3	0.00	0.00	0
Progne_tapera	0.11	0.66	1	0.00	0.00	0	0.31	1.41	2	0.00	0.00	0
Stelgidopterix_serripennis	0.00	0.00	0	0.00	0.00	0	0.97	5.83	1	0.00	0.00	0
Pygochelidon_cyanoleuca	3.76	9.64	12	1.46	3.80	8	1.25	2.49	9	1.14	2.23	10
Tachycineta_albiventer	0.00	0.00	0	0.14	0.82	1	0.00	0.00	0	0.00	0.00	0
Tachycineta__leucopyga	0.05	0.33	1	0.05	0.33	1	0.58	2.16	4	0.00	0.00	0
Tachycineta_leucorrhoa	0.49	1.43	5	0.00	0.00	0	0.22	0.90	3	0.00	0.00	0
Corthylio_calendula	0.00	0.00	0	0.14	0.48	3	0.03	0.17	1	0.08	0.28	3
Psaltriparus_minimus	0.24	0.93	3	0.05	0.23	2	0.36	1.44	3	0.22	1.33	1
Troglodytes_aedon	1.27	2.24	18	1.51	2.06	23	3.33	4.93	22	2.86	4.45	21
Thryomanes_bewickii	0.27	0.84	4	0.35	1.25	3	0.64	1.93	4	0.28	0.81	4
Campylorhynchus_zonatus	0.14	0.82	1	0.16	0.99	1	0.44	2.67	1	0.14	0.83	1
Polioptila_caerulea	0.00	0.00	0	0.16	0.44	5	0.00	0.00	0	0.25	1.34	2
Polioptila_dumicola	0.03	0.16	1	0.16	0.99	1	0.03	0.17	1	0.00	0.00	0
Turdus_flavipes	0.03	0.16	1	0.11	0.52	2	0.00	0.00	0	0.00	0.00	0
Turdus_leucomelas	0.38	1.06	5	0.41	1.19	4	0.11	0.67	1	0.14	0.54	3
Turdus_rufiventris	4.73	10.85	10	3.08	6.70	11	3.50	7.79	9	3.78	9.39	8
Turdus_grayi	0.62	2.74	3	0.35	1.49	2	0.39	1.25	4	0.28	1.21	3
Turdus_migratorius	0.35	1.09	4	0.03	0.16	1	0.25	0.81	4	0.14	0.49	3
Turdus_rufopalliatus	0.92	2.89	4	0.16	0.99	1	0.22	0.83	3	0.11	0.67	1
Turdus__falcklandii	5.86	18.80	4	8.62	27.73	4	3.17	9.71	4	2.89	10.71	4
Turdus_amaurochalinus	0.68	2.30	5	0.22	1.16	2	0.44	1.46	4	0.22	0.76	4
Turdus_subalaris	0.05	0.33	1	0.00	0.00	0	0.03	0.17	1	0.00	0.00	0
Turdus_ignobilis	0.03	0.16	1	0.00	0.00	0	0.00	0.00	0	0.00	0.00	0
Turdus_chiguanco	3.43	10.71	5	2.14	6.52	5	1.56	5.61	4	1.78	5.69	5
Ptiliogonys_cinereus	0.11	0.66	1	0.32	1.97	1	0.03	0.17	1	0.11	0.52	2
Estrilda_astrild	0.00	0.00	0	0.22	1.32	1	0.03	0.17	1	0.11	0.52	2
Passer_domesticus	5.54	7.67	22	4.05	7.88	18	4.78	8.27	19	4.06	8.76	18
Sturnus_vulgaris	0.49	2.34	3	0.46	2.18	3	1.25	5.80	2	0.72	2.59	3
Melanotis_caerulescens	0.00	0.00	0	0.00	0.00	0	0.03	0.17	1	0.00	0.00	0
Dumetella_carolinensis	0.03	0.16	1	0.00	0.00	0	0.06	0.33	1	0.11	0.67	1
Toxostoma_curvirostre	0.00	0.00	0	0.03	0.16	1	0.06	0.33	1	0.03	0.17	1
Mimus__thenca	0.00	0.00	0	0.00	0.00	0	0.06	0.33	1	0.03	0.17	1
Mimus_saturninus	0.95	2.88	5	1.08	2.89	6	2.17	7.14	6	1.25	5.30	5
Anthus_lutescens	0.08	0.49	1	0.00	0.00	0	0.00	0.00	0	0.00	0.00	0
Basileuterus_culicivorus	0.19	0.66	3	0.24	0.89	4	0.00	0.00	0	0.00	0.00	0
Basileutrus_bivittatus	0.00	0.00	0	0.05	0.33	1	0.00	0.00	0	0.00	0.00	0
Myiothlypis_leucoblephara	0.24	0.86	3	0.16	0.55	3	0.00	0.00	0	0.00	0.00	0
Cardellina_pusilla	0.03	0.16	1	0.35	1.40	3	0.06	0.33	1	0.75	3.52	4
Myioborus_brunniceps	0.00	0.00	0	0.08	0.49	1	0.00	0.00	0	0.00	0.00	0
Mniotilta_varia	0.00	0.00	0	0.03	0.16	1	0.00	0.00	0	0.03	0.17	1

Leiothlypis_celata	0.05	0.33	1	0.00	0.00	0	0.00	0.00	0	0.00	0.00	0
Leiothlypis_ruficapilla	0.14	0.67	2	0.14	0.82	1	0.08	0.37	2	0.00	0.00	0
Geothlypis_aequinoctialis	0.00	0.00	0	0.00	0.00	0	0.00	0.00	0	0.03	0.17	1
Setophaga_pitiayumi	0.22	0.75	3	0.16	0.73	2	0.22	0.76	3	0.08	0.37	2
Setophaga_petechia	0.16	0.60	3	0.03	0.16	1	0.00	0.00	0	0.06	0.33	1
Setophaga_coronata	0.19	0.74	3	0.70	2.81	4	0.06	0.23	2	0.22	0.83	3
Setophaga_nigrescens	0.00	0.00	0	0.00	0.00	0	0.00	0.00	0	0.06	0.33	1
Setophaga_townsendi	0.00	0.00	0	0.08	0.36	2	0.00	0.00	0	0.08	0.37	2
Setophaga_virens	0.00	0.00	0	0.14	0.82	1	0.00	0.00	0	0.17	1.00	1
Coereba_flaveola	0.78	1.93	8	1.08	3.33	5	0.92	3.09	4	0.75	2.42	4
Conirostrum_cinereum	0.35	1.60	2	0.03	0.16	1	0.36	1.36	3	0.11	0.52	2
Diglossa_baritula	0.03	0.16	1	0.00	0.00	0	0.06	0.33	1	0.00	0.00	0
Diglossa_carbonaria	0.19	0.81	2	0.14	0.82	1	0.08	0.50	1	0.08	0.50	1
Dacnis_cayana	0.08	0.49	1	0.08	0.49	1	0.00	0.00	0	0.00	0.00	0
Euphonia_chlorotica	0.19	0.66	3	0.46	1.37	5	0.33	1.53	3	0.44	2.67	1
Euphonia_violacea	0.03	0.16	1	0.00	0.00	0	0.00	0.00	0	0.00	0.00	0
Euphonia_affinis	0.00	0.00	0	0.00	0.00	0	0.03	0.17	1	0.00	0.00	0
Euphonia_elegantisima	0.00	0.00	0	0.00	0.00	0	0.00	0.00	0	0.03	0.17	1
Euphonia_hirundinacea	0.00	0.00	0	0.00	0.00	0	0.06	0.33	1	0.17	1.00	1
Haemorhous_mexicanus	0.84	2.46	4	0.54	2.53	3	0.89	2.78	5	1.14	4.73	3
Spinus_psaltria	0.08	0.36	2	0.11	0.46	2	0.36	1.42	3	0.08	0.28	3
Spinus_xanthogastrus	0.00	0.00	0	0.08	0.49	1	0.11	0.67	1	0.00	0.00	0
Spinus_magellanica	0.32	1.11	5	0.35	1.34	3	0.72	2.09	8	0.28	1.50	2
Spinus_atratus	0.19	1.15	1	0.14	0.59	2	0.36	2.00	2	0.06	0.33	1
Spinus__barbatus	0.00	0.00	0	0.00	0.00	0	0.28	1.67	1	0.00	0.00	0
Chlorospingus_flavopectus	0.00	0.00	0	0.00	0.00	0	0.11	0.67	1	0.00	0.00	0
Rauenia_bonariensis	0.00	0.00	0	0.14	0.82	1	0.03	0.17	1	0.08	0.50	1
Thraupis_sayaca	3.41	7.00	14	2.86	6.49	12	2.53	5.61	11	0.97	2.81	9
Thraupis_palmarum	0.08	0.36	2	0.11	0.39	3	0.14	0.59	2	0.11	0.67	1
Thraupis_abbas	0.16	0.99	1	0.00	0.00	0	0.19	1.17	1	0.00	0.00	0
Thraupis_episcopus	0.43	1.50	3	0.57	2.06	3	0.17	1.00	1	0.31	1.19	3
Piranga_flava	0.00	0.00	0	0.00	0.00	0	0.06	0.33	1	0.03	0.17	1
Piranga_rubra	0.00	0.00	0	0.14	0.42	4	0.00	0.00	0	0.06	0.23	2
Piranga_ludoviciana	0.03	0.16	1	0.03	0.16	1	0.00	0.00	0	0.00	0.00	0
Phrygilus_punensis	0.27	1.15	2	0.35	1.49	2	0.31	1.41	2	0.31	1.83	1
Sicalis_flaveola	1.03	2.69	7	0.86	2.99	6	0.69	1.60	7	0.89	2.53	6
Sicalis_luteola	0.16	0.69	2	0.00	0.00	0	0.06	0.33	1	0.31	1.83	1
Sicalis_uropygialis	0.00	0.00	0	0.00	0.00	0	0.11	0.67	1	0.00	0.00	0
Sicalis_olivascens	0.00	0.00	0	0.00	0.00	0	0.42	2.50	1	0.14	0.83	1
Spizella_passerina	0.00	0.00	0	0.03	0.16	1	0.00	0.00	0	0.00	0.00	0
Zonotrichia_capensis	4.03	7.13	19	3.03	6.90	15	7.50	14.07	20	7.39	15.49	17
Aimophila_rufescens	0.00	0.00	0	0.00	0.00	0	0.14	0.83	1	0.00	0.00	0
Melozone_fusca	0.35	1.30	3	0.05	0.33	1	0.14	0.68	2	0.11	0.67	1
Paroaria_coronata	0.38	1.75	2	0.22	0.82	3	0.11	0.67	1	0.03	0.17	1
Thlypopsis_ruficeps	0.00	0.00	0	0.03	0.16	1	0.00	0.00	0	0.00	0.00	0
Microspingus_cabanisi	0.00	0.00	0	0.00	0.00	0	0.00	0.00	0	0.03	0.17	1

Microspingus_melanoleucus	0.00	0.00	0	0.05	0.33	1	0.00	0.00	0	0.00	0.00	0
Volatinia_jacarina	0.00	0.00	0	0.05	0.33	1	0.69	3.54	2	0.17	1.00	1
Sporophila_caerulescens	0.00	0.00	0	0.03	0.16	1	0.00	0.00	0	0.00	0.00	0
*Diuca__diuca_*	3.57	21.70	1	0.19	1.15	1	3.06	13.06	3	0.00	0.00	0
Pheucticus_ludovicianus	0.03	0.16	1	0.00	0.00	0	0.00	0.00	0	0.00	0.00	0
Pheucticus_melanocephalus	0.14	0.54	3	0.00	0.00	0	0.11	0.46	2	0.00	0.00	0
Saltator_coerulescens	0.19	0.81	2	0.05	0.33	1	0.11	0.40	3	0.00	0.00	0
Saltator_aurantiirostris	0.08	0.36	2	0.00	0.00	0	0.19	1.01	2	0.03	0.17	1
Saltator_similis	0.08	0.36	2	0.00	0.00	0	0.11	0.46	2	0.00	0.00	0
Saltator_atriceps	0.00	0.00	0	0.00	0.00	0	0.08	0.50	1	0.00	0.00	0
Leistes_loyca	0.00	0.00	0	0.00	0.00	0	0.42	1.78	2	0.58	2.89	2
Psarocolius_montezuma	0.00	0.00	0	0.00	0.00	0	0.00	0.00	0	0.08	0.37	2
Cacicus_haemorrous	0.00	0.00	0	0.05	0.33	1	0.00	0.00	0	0.00	0.00	0
Icterus_spurius	0.00	0.00	0	0.00	0.00	0	0.03	0.17	1	0.00	0.00	0
Icterus_pyrrhopterus	0.00	0.00	0	0.05	0.23	2	0.08	0.37	2	0.17	1.00	1
Icterus_graduacauda	0.00	0.00	0	0.00	0.00	0	0.06	0.33	1	0.00	0.00	0
Icterus_galbula	0.05	0.33	1	0.03	0.16	1	0.11	0.67	1	0.03	0.17	1
Icterus_abeillei	0.14	0.59	2	0.03	0.16	1	0.14	0.54	3	0.06	0.23	2
Molothrus_rufoaxillaris	0.49	2.50	2	0.00	0.00	0	0.56	2.37	4	0.06	0.33	1
Molothrus_bonariensis	2.95	5.98	15	1.65	5.17	10	2.67	5.39	14	2.97	9.10	6
Molothrus_aeneus	0.00	0.00	0	0.00	0.00	0	0.11	0.46	2	0.00	0.00	0
Dives_dives	0.03	0.16	1	0.03	0.16	1	0.08	0.37	2	0.03	0.17	1
Curaeus__curaeus	0.24	0.93	3	0.19	1.00	2	0.75	2.67	3	0.50	2.83	2
Quiscalus_mexicanus	2.11	6.53	7	1.81	5.75	7	2.19	10.74	4	0.83	3.34	5
Agelaioides_badius	0.62	2.13	5	0.16	0.83	2	0.56	2.36	5	0.36	1.55	2
Chrysomus_ruficapillus	0.59	3.62	1	0.70	4.27	1	0.00	0.00	0	0.00	0.00	0
